# Motor Control and Ergonomic Intervention Home-Based Program: A Pilot Trial Performed in the Framework of the Motor Control Home Ergonomics Elderlies' Prevention of Falls (McHeELP) Project

**DOI:** 10.7759/cureus.14336

**Published:** 2021-04-07

**Authors:** Sophia Stasi, Maria Tsekoura, John Gliatis, Vasiliki Sakellari

**Affiliations:** 1 Physiotherapy, Physiotherapy Department, Faculty of Health and Care Sciences, University of West Attica (UNIWA), Athens, GRC; 2 Physiotherapy, Physiotherapy Department, School of Health Rehabilitation Sciences, University of Patras, Patras, GRC; 3 Orthopaedics, Department of Medicine, School of Health Rehabilitation Sciences, University of Patras, Patras, GRC

**Keywords:** older adults, falls, lower limb, physiotherapy, home-based programme, motor control, ergonomics

## Abstract

Objectives

Falls are a serious problem that can reduce living autonomy and health-related quality of life of older adults. A decrease in the muscular strength of the lower limbs and the deterioration of balance or motor performance deficits may lead to falls. "Motor Control Home Ergonomics Elderlies' Prevention of Falls" (McHeELP) is a novel motor control exercise program combined with ergonomic arrangements of the home environment. This pilot trial is conducted in order to examine the feasibility and acceptability of the McHeELP program, the selection of the most appropriate outcome measures, and the exact sample size calculation that should be used for the randomized controlled trial (RCT) with Clinical Trial Identifier: ISRCTN15936467.

Patients and methods

Twenty older adults (aged ≥65 years) who had experienced at least one fall-incident in the past 12 months have participated in the trial; they were randomized in a 1:1 ratio to the McHeELP group (McHeELP-G) and the Control group (CG). The McHeELP-G received a personalized therapeutic motor control and learning exercise program performed three times per week for 12 weeks. Regarding McHeELP - home modification, a booklet that contained basic advice and tips on the modification for their inside and outside home environment was provided to the participants. Objective and self-reported outcome measures, collected at baseline and post-intervention (end of the third month), included functional, fear of falling, and quality of life measurements.

Results

The McHeELP intervention was very feasible and acceptable to the participants, and the adherence was excellent (100%). The majority of outcome measures seemed appropriate and significant differences were also revealed between the two groups. Specifically, post-intervention statistically significant improvement was found in the 4 meters walking test, Timed Up and Go test, Sit to Stand test, Tandem Stance test, Functional Reach test, Foot tapping test, EuroQoL-5D-5L - visual analog scale (VAS), Lower Extremity Functional Scale, Falls Self-Efficacy International Scale, and Home Falls and Accidents Screening Tool (HOMEFAST) questionnaire of McHeELP-G (all p-values ≤0.002). No statistically significant difference was observed in the mobility, self-care, usual activities, pain/discomfort subscales of Euro QoL-5D-5L (all p-values >0.05), except the anxiety/depression subscale of McHeELP-G (p=0.008). Moreover, no statistically significant improvement was found regarding McHeELP participants' knee flexion/extension restriction and ankle dorsiflexion/plantar-flexion restrictions. Regarding CG, no statistically significant difference was found (p>0.05), except the Tandem Stance test (p=0.003) and HOMEFAST (p<0.001). Referring to the future McHeELP RCT, it was estimated that a sample size of 25 evaluable patients per group is required.

Conclusions

This pilot trial's findings suggest that it is feasible to deliver an RCT of the McHeLP program to this population. Exercise programs that are easy to administer need to be developed and implemented to reduce the burden of falls in older adults.

## Introduction

As the proportion of older adults is increasing worldwide, their health is becoming an important issue [1]. The age-related decline in movement and/or functional capacity negatively affects the self-conﬁdence of older adults, hampering the performance of activities of daily living (ADLs) due to fear of falling or other physical, psychological, or social factors [2]. Moreover, these restrictions may progressively compromise functional autonomy over time, creating an irreversible cycle, resulting in partial or total dependence [2]. Falls are a severe problem that can reduce living autonomy and health-related quality of life (QoL) of older adults [1]. Any level of decrease in the muscular strength of the lower limbs, the deterioration of balance [3], or motor performance deficits may lead to falls [4].

Motor control is defined as the process of initiating, directing, and grading purposeful voluntary movement [5]. Motor performance deficits in older adults include difficulty in coordination, increased variability, and slowing of movement, as well as balance and gait difficulties [5]. Motor learning is the process of acquiring a skill by which the patient, through practice and assimilation, refines and makes automatic the desired movement while training specificity is the key element of motor learning [4]. Research indicates that exercise is an efficacious intervention for fall prevention among older adults [6]. On the other hand, older adults spend most of their time in their resident (home) because they feel safer. However, most falls resulting in fragility fractures and other injuries may occur at home [6]. Therefore, reducing the home-living environmental hazards is an essential contributor to the successful maintenance of older adult's well-being. It was reported that environmental limitations might lead to negative outcomes such as accidental falls and home-related injuries [7]. However, little is known regarding motor control exercises for preventing falls, and information on home interventions is inconsistent [7].

The "Motor Control Home Ergonomics Elderly Prevention of Falls" (McHeELP) is a home-based motor control exercise program combined with ergonomic arrangements of the home environment. The McHeELP exercise program concept was based on the most common approaches used in motor learning research, namely, the Schmidt classification and the Cratty classification regarding gross motor skills [8]. It was hypothesized that the McHeELP program would improve functionality and reduce possible fall-risk factors in the home environment and the fear of falling in older adults. This pilot trial is conducted in order to examine the feasibility of the McHeELP protocol, the acceptability of the intervention, the selection of the most appropriate primary outcome measure, and the exact sample size calculation that should be used for the main study (a randomized controlled trial (RCT) with Clinical Trial Identifier: ISRCTN15936467).

## Materials and methods

Ethics

The present two-group pilot trial was performed in the McHeELP project's framework, in accordance with the ethical principles stated in the Declaration of Helsinki and its later amendments [9]. The Ethics Committee of the University of Patras, Greece, approved the study protocol (No: 9807/05/02/2020). The study conformed to the "Consolidated Standards of Reporting Trials" (CONSORT) extension statement for randomized pilot and feasibility trials [10].

Participants

Participants were recruited from the regions of Greece, Attica, and Achaia using flyers, posters, and advertisements. Prior to recruitment, participants were screened for eligibility to meet the following inclusion criteria: aged ≥65 years old and experienced at least one fall-incident in the past 12 months. Participants were excluded if they have been diagnosed with cognitive impairment, lower-limb muscle weakness due to a central or peripheral neurological etiology, have been told not to exercise by a physician, were already participating in an exercise program, or if they were suffered from a vision, vestibular, or other medical problem that could affect their ability to complete objective assessments and perform the exercise program. Upon acceptance, participants gave their written informed consent, and their demographic and clinical characteristics were recorded.

Randomization

After receiving written informed consent from the participant, they were allocated into either the intervention group (McHeELP-G) or the control group (CG). The 1:1 randomization process was used for the McHeELP pilot study because this allocation ratio provides the greatest power for testing effectiveness for the future RCT [10]. The participants were blinded to group allocation.

Interventions

A 12-week home-based motor control exercise program combined with an ergonomic home modification was implemented in the McHeELP group. The McHeELP exercise program includes a package of motor control exercises, which are divided into six domains, namely, "Warm up," "Serial skills," "Cognitive skills," "Balance," "Sensory strategy," and "Dynamic control." The exercises of the program are described in detail in the research protocol of this project [8]. McHeELP-G participants received a personalized exercise program (two exercises in each domain; a total of 12 exercises), which were performed three times per week for a 12-week time period. Depending on the participant's ability, each session lasted for at least 20 min but not above the 30 min mark. During day one of the first week of the McHeELP program, the physiotherapist provided the participant with a health and wellness education session and their individualized McHeELP program while also training them on how to perform exercises safely and correctly. During the 12-week period, the physiotherapist revisited each participant and revised the individuated program, at three more time-points (at the end of the first, fourth, and eighth weeks), so that to make corrections and progressive adjustments to the exercises. During each visit trunk muscles, posture, movement pattern, and breathing were assessed and corrected by the physiotherapists. Additionally, participants were asked to keep an exercise diary and record the days they completed the program and the room temperature selected for exercising [8]. If it was rendered necessary, participants were prompted to ask for over-the-phone guidance.

The older adults that were allocated in the CG received no exercises during the time of the study. This group received the same health and wellness education session as the McHeELP-G. Participants of both groups were instructed not to engage in additional exercising for the period of 12 weeks.

Regarding the McHeELP-home modification part, on the baseline session, a booklet, which included basic advice and tips on modifying the interior and exterior of their home was provided to the participants in both groups. The adjustments were of low costs, such as removing carpets that were either worn out or with loose/deep piles, moving furnishings to create clear paths, fix slippery surfaces, replace lamps with insufficient lighting, etc. [7]. The key difference between the study's groups was that during the three intermediate appointment sessions, the participants of McHeELP-G were reminded to materialize these modifications. In contrast, the participants of CG were merely advised on their baseline session or appointment.

Outcomes and procedures

Each participant was interviewed face-to-face to assess their history of falls, lifestyle habits, exercise habits, and medication use, and information on meal frequency, smoking, and drinking statuses were also obtained. Demographic variables, including age, gender, living status, and educational level, were also recorded. In order to verify cognition state changes at admission or to detect any signs of undiagnosed cognitive impairment, participants also completed the Mini-Cog test (v.01.19.16; Mini-Cog™ © S. Borson) [8].

In the present study, participants were assessed at baseline and immediately post-intervention (end of the third month) with the physical performance measures (PPMs): 4 meters walking test [11], Timed Up and Go (TUG) test [12], Sit to Stand test - 30 seconds [13], Tandem Stance (heel-toe) test (Tandem) [14], Functional Reach Test (FRT) [15], Foot Tapping test - 10 seconds [16], Heel to Shin test [17]; and the Greek versions of the patient-reported outcomes (PROs): Euro QoL-5D-5L questionnaire (EQ-5D-5L) [18], Lower Extremity Functional Scale (LEFS-Greek) [19], Falls Self-Efficacy International Scale (FES-I_GREEK) [20], and the Home Falls and Accidents Screening Tool (HOMEFAST) questionnaire [21]. The description and procedures of the study's outcome measures are presented in the Appendix. Additionally, the knee flexion/extension and ankle dorsiflexion/plantarflexion restrictions were measured as a qualitative clinical outcome. As restriction was defined as any range of motion below the normal that could affect the performance of either the exercises or the PPMs [22].

The needed equipment for the performance of the objective PPMs was portable, so the measurements data were collected at the home of the participants, by two examiners, one for Attica and one for the Achaia groups. The examiners were independent and not aware of participants' randomization.

Statistical analysis

Twenty older adults participated in the present pilot trial because it is recommended that for standardized effect sizes above 0.8, a pilot trial sample size of 10 participants per group is required [23].

Data were expressed as mean ± SD for quantitative variables and as percentages for qualitative variables. The Kolmogorov-Smirnov test was utilized for normality analysis of the quantitative variables. The homogeneity between compared groups was examined using the student's t-test, the chi-squared test, and Fisher's exact test for the quantitative and qualitative demographic/clinical variables and outcome measures, respectively. The intervention's efficacy during the observation period was evaluated by calculating the mean percentage changes from baseline to post-intervention (end of the third month). Comparisons of percentage change from baseline of outcome measures during the observation period between the two groups were analyzed using the independent samples t-test or Mann-Whitney tests in case of violation of normality. The comparisons of change of categorical variables from baseline to 3rd month, between groups, were examined using the chi-square test. All tests were two-sided and a p-value of <0.05 was used to denote statistical significance. All analyses were carried out using the Statistical Package for the Social Sciences (SPSS) version 21.00 (IBM Corporation, Armonk, NY).

The sample size for the main study was estimated using G*Power version 3.1.9.2. We set two outcome variables (TUG test and/or Tandem test) and the sample size calculation was based on the percent change from baseline to the end of intervention (end of the third month) of these variables.

## Results

**Figure 1:**
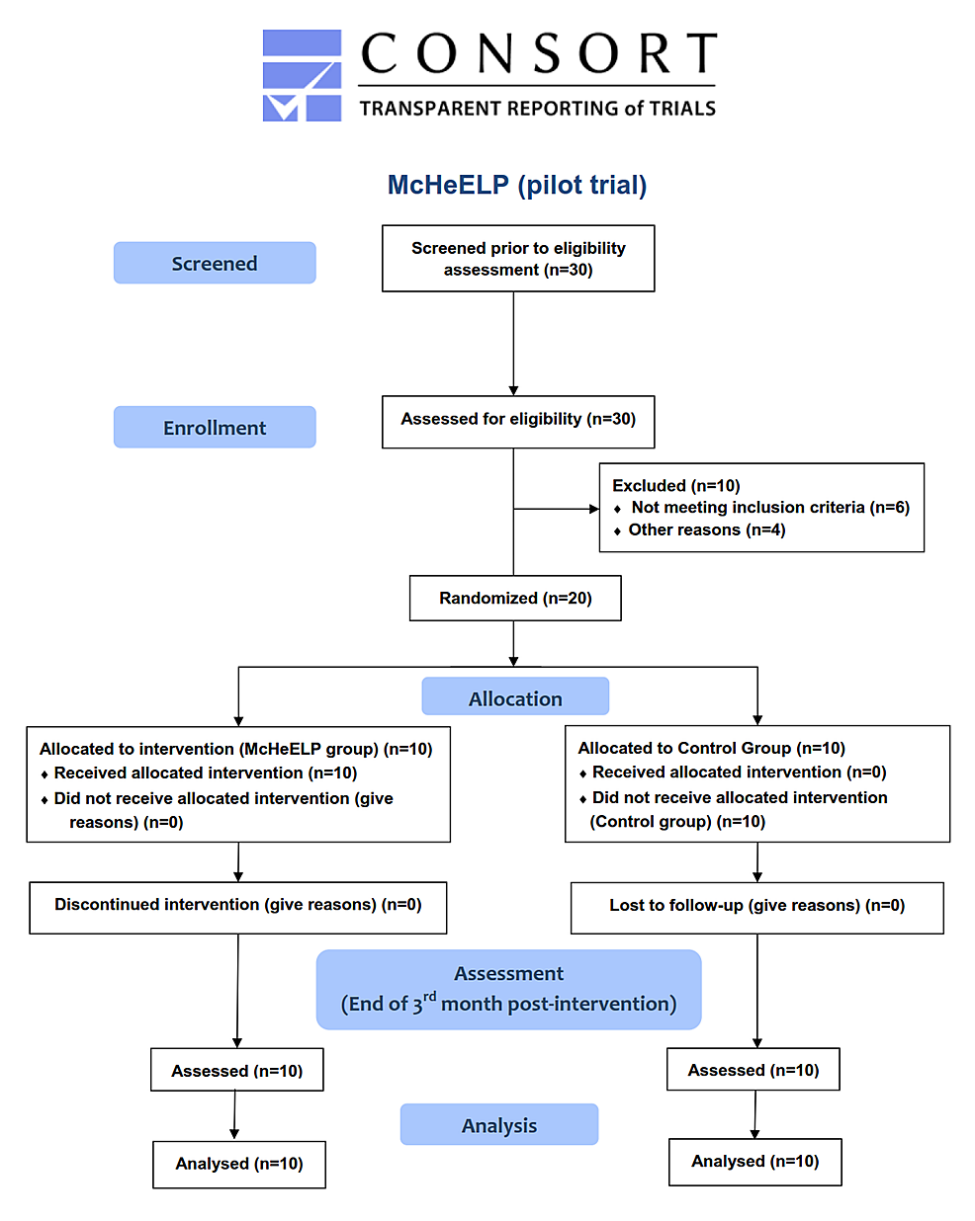
Flow diagram of the McHeELP pilot trial McHeELP: Motor Control Home Ergonomics Elderlies' Prevention of Falls

Patient recruitment lasted from May to July 2020, by which time the required number of participants had been reached. The recruitment procedure is depicted in the flow diagram in Figure 1. Demographic, habitual, and clinical characteristics at baseline are shown in Table 1 while clinical quantitative and qualitative outcomes are shown in Table 2. Non-significant differences were found between McHeELP-G and CG regarding the participants' personal and clinical characteristics, as well as all clinical outcomes at baseline (all p-values >0.05).

**Table 1:** Comparison of patients’ characteristics at baseline Significant p-value < 0.05 * The values are presented as mean ± standard deviation yrs=years; m=metres; kg=kilograms; McHeELP: Motor Control Home Ergonomics Elderlies' Prevention of Falls

Characteristics	McHeLP Group (n=10)	Control Group (n=10)	p-value
Age (yrs)*	79.4±5.27	76.4±6.03	0.870
Gender (n, %)			
Women	4 (40%)	4 (40%)	1.00
Men	6(60%)	6 (60%)	
Height (m)*	1.62.3 ± 6.23	1.62.6 ±.6.05	0.910
Weight (kg)*	77.3. ±.10.1.	76.6. ±9.17	0.870
ΒΜΙ (kg/m^2^)*	24.6±2.6	23.7±4	0.700
Number of used drugs*	4.1±0.87	4±0.66	0.770
Number of comorbidities*	4.4±1.07	4.2±1.1	0.670
Incidents of fall (last 12 months)	1.5±0.7	1.6±0.69	0.750
Meals per day	2.2±0.42	2.3±0.48	0.620
Smoking (n, %)			
Yes	1 (10%)	1 (10%)	1.00
No	9 (90%)	9 (90%)	
Alcohol (n, %)			
Yes every day	0 (0%)	0 (0%)	0.620
Yes occasionally	2 (20%)	3 (30%)	
No	8 (80%)	7 (70%)	
Sleep (n, %)			
4-6 hours	0 (0%)	0 (0%)	0.620
7-8 hours	7 (70%)	8 (80%)	
9-10 hours	3 (20%)	2 (20%)	
Education (n, %)			
Illiterate	0 (0%)	0 (0%)	0.790
Elementary	3 (30%)	3 (30%)	
High school	3 (30%)	4 (4035%)	
University	4 (40%)	3 (30%)	
Marital status (n, %)			
Single	0 (0%)	0 (0%)	1.00
Married	3 (30%)	63 (30%)	
Divorced	0 (0%)	0 (0%)	
Widowed	7 (70%)	7 (70%)	
Living alone (n, %)			
Yes	3 (30%)	2 (20%)	0.620
Νο	7 (60%)	8 (80%)	
Walking aids (n, %)			
Yes	3 (30%)	2 (20%)	0.620
Νο	7 (70%)	8 (80%)	
Mini-Cog test (v.01.19.16) (n, %)			
0 (Positive for cognitive impairment)	0 (0%)	0 (0%)	1.00
1 (Positive for cognitive impairment)	0 (0%)	0 (0%)	
2 (Positive for cognitive impairment)	0 (0%)	0 (0%)	
3 (Need thorough evaluation)	0 (0%)	0 (0%)	
4 (Negative for cognitive impairment)	0 (0%)	0 (0%)	
5 (Negative for cognitive impairment)	10 (100%)	10 (100%)	

**Table 2:** Comparison of clinical outcomes between groups at baseline Significant p-value < 0.05 * The values are presented as mean ±standard deviation McHeELP: Motor Control Home Ergonomics Elderlies' Prevention of Falls

Clinical variables	McHeLP group (n=10)	Control Group (n=10)	p-value
4 meters walking test* (s)	3.95 ±0.94	4.08 ±0.80	0.749
Timed Up & Go test* (s)	12.25 ±.56	12.49 ±1.43	0.723
Sit-to-stand test* (reps)	13.40 ±3.03	12.90 ±2.64	0.700
Foot taping test-right* (reps)	24.60 ±4.06	23.60 ±4.35	0.602
Foot taping test-left* (reps)	24.30 ±4.24	24.40 ±4.35	0.959
Tandem stance test (heel-toe)* (s)	21.45 ±3.79	22.00 ±4.76	0.778
Functional reach test – right* (cm)	25.70 ±2.45	26.60 ±2.67	0.443
Functional reach test – left* (cm)	25.40 ±2.32	26.25 ±2.32	0.424
Heel to shin test (right heel to left shin)*	0.00 ±0.00	0.00 ±0.00	–
Heel to shin test (left heel to right shin)*	0.00 ±0.00	0.00 ±0.00	–
Knee flexion restriction (n, %)			
Yes	4 (40.0%)	0 (0.0%)	0.082
No	6 (60.0%)	10 (100.0%)	
Knee extension restriction (n, %)			
Yes	2 (20.0%)	2 (20.0%)	1.00
No	8 (80.0%)	8 (80.0%)	
Ankle dorsiflexion restriction (n, %)			
Yes	2 (20.0%)	2 (20.0%)	1.00
No	8 (80.0%)	8 (80.0%)	
Ankle plantar flexion restriction (n, %)			
Yes	0 (0.0%)	0 (0.0%)	1.00
No	10 (100.0%)	10 (100.0%)	
Euro QoL-5D-5L – Mobility subscale (n, %) (min=1, max=5)			
2: I have slight problems in walking about	1 (10.0%)	1 (10.0%)	0.819
3: I have moderate problems in walking about	8 (80.0%)	7 (70.0%)	
4: I have severe problems in walking about	1 (10.0%)	2 (20.0%)	
Euro QoL-5D-5L – Self-care subscale (n, %) (min=1, max=5)			
1: I have no problems washing or dressing myself	6 (60.0%)	4 (40.0%)	0.645
2: I have slight problems washing or dressing myself	3 (30.0%)	4 (40.0%)	
3: I have moderate problems washing or dressing myself	1 (10.0%)	2 (20.0%)	
Euro QoL-5D-5L – Usual activities subscale (n, %) (min=1, max=5)			
1: I have no problems doing my usual activities	1 (10.0%)	0 (0.0%)	0.580
2: I have slight problems doing my usual activities	4 (40.0%)	4 (40.0%)	
3: I have moderate problems doing my usual activities	5 (50.0%)	6 (60.0%)	
Euro QoL-5D-5L – Pain/Discomfort subscale (n, %) (min=1, max=5)			
2: I have slight pain or discomfort	3 (30.0%)	4 (40.0%)	1.000
3: I have moderate pain or discomfort	7 (70.0%)	6 (60.0%)	
Euro QoL-5D-5L – Anxiety/Depression subscale (n, %) (min=1, max=5)			
1: I am not anxious or depressed	1 (10.0%)	0 (0.0%)	0.284
2: I am slightly anxious or depressed	1 (10.0%)	4 (40.0%)	
3: I am moderately anxious or depressed	5 (50.0%)	5 (50.0%)	
4: I am severely anxious or depressed	3 (30.0%)	1 (10.0%)	
Euro QoL-5D-5L – VAS* (100%=best health)	70.00 ±10.00	71.50 ±14.15	0.787
Lower Extremity Functional Scale – Greek version* [max =80 (very high functionality)]	40.60 ±9.66	39.20 ±8.43	0.734
Falls Efficacy Scale – International_GREEK* [max=64 (severe fear of falling)]	43.50 ±9.73	39.60 ±10.83	0.408
Home Falls and Accidents Screening Tool* [max=25 (higher risk of falling within home environment]	3.10 ±1.45	2.90 ±1.20	0.740

The values of the post-intervention vs baseline difference of all quantitative variables between groups are shown in Table 3. Post-intervention, a statistically significant improvement was found in the 4 meters walking test, TUG test, Sit to Stand test (30 seconds), Tandem test, Functional Reach test, Foot Tapping test (10 seconds), Euro QoL-5D-5L - visual analog scale (VAS), Lower Extremity Functional Scale (LEFS-Greek), Falls Self-Efficacy International Scale (FES-I_GREEK), and the HOMEFAST of McHeELP-G (all p-values ≤0.002). No statistically significant difference was found in the Control group's quantitative outcomes (p>0.05), except the Tandem Stance test (p=0.003) and HOMEFAST (p<0.001) (Table 3).

**Table 3:** Comparison of clinical quantitative outcomes’ measurements between groups Significant p-value < 0.05 * Post-intervention= end of 3rd month (13th week post-intervention) **NE= not evaluable; s=seconds; reps=repetitions; cm=centimetres McHeELP: Motor Control Home Ergonomics Elderlies' Prevention of Falls

	McHeLP group (n=10)				Control group (N=10)			
Clinical quantitative variables	Baseline (mean±SD)	Post-intervention * (mean±SD)	Post-intervention* vs baseline difference (mean[95%CI])	p-value	Baseline (mean±SD)	Post-intervention (mean±SD)	Post-intervention* vs baseline difference (mean[95%CI])	p-value
4 meters walking test (s)	3.95±0.94	3.07±0.60	-0.88[-0.58 -1.19]	<0.001	4.08±0.80	4.03±0.78	-0.05[-0.37 0.47]	0.795
Timed Up & Go test (s)	12.25±1.56	8.08 ±1.45	-4.17[-3.75 -4.60]	<0.001	12.49±1.43	12.49±1.48	0.00[-0.54 0.54]	1.000
Sit-to-stand test (30-s/reps)	13.40±3.03	16.80±2.90	3.40[2.90 3.90]	<0.001	12.90±2.64	12.90±3.35	0.00[-1.62 1.62]	1.000
Foot tapping test-right (10-s/reps)	24.60±4.06	32.30±5.48	7.7 [5.65 9.75]	<0.001	23.60±4.35	23.20±4.57	0.40[-0.73 1.53]	0.443
Foot tapping test-left (10-s/reps)	24.30±4.24	32.50±5.48	8.20[5.92 10.48]	<0.001	24.40±4.35	23.90±4.23	0.50[-2.07 3.57]	0.671
Tandem stance test (heel-toe) (s)	21.45±3.79	28.10±3.73	6.65[5.10 8.20]	<0.001	22.00±4.76	24.80±3.74	2.80[1.26 4.34]	0.003
Functional reach test – right (cm)	25.70±2.45	35.00±3.02	9.30[6.73 11.87]	<0.001	26.60±2.67	27.50±3.27	0.90[-1.99 0.19]	0.100
Functional reach test – left (cm)	25.40±2.32	35.30±3.74	9.90[7.19 12.61]	<0.001	26.25±2.32	27.20±3.08	0.95[0.12 2.02]	0.076
Heel to shin test (right heel to left shin)	0.00±0.00	0.00±0.00	–	NE**	0.00±0.00	0.00±0.00	–	NE**
Heel to shin test (left heel to right shin)	0.00±0.00	0.00±0.00	–	NE**	0.00±0.00	0.00±0.00	–	NE**
Euro QoL-5D-5L – VAS (100%=best health)	70.00±10.00	77.50±8.25	7.50[4.46 10.54]	<0.001	71.50±14.15	73.00±12.95	1.50[2.29 5.29]	0.394
Lower Extremity Functional Scale – Greek version [max =80 (very high functionality)]	40.60±9.66	43.40±9.56	2.80[0.67 4.93]	0.016	39.20±8.43	38.80±8.52	-0.40[-0.10 -0.90]	0.104
Falls Efficacy Scale – International_GREEK [max=64 (severe concerns about fall-related self-efficacy)]	43.50±9.73	35.40±6.29	-8.10[-4.03 -12.17]	0.001	39.60±10.83	40.00±10.74	0.40[1.57 2.37]	0.657
Home Falls and Accidents Screening Tool [max=25 (higher risk of falling within the home environment)]	3.10±1.45	1.20±0.79	-1.90[-0.86 -2.94]	0.002	2.90±1.20	0.80±0.63	-2.10[-1.47 -2.73]	<0.001

Post-intervention, no statistically significant improvement was found regarding McHeELP-G's knee flexion/extension restriction, ankle dorsiflexion/plantarflexion restrictions, Euro QoL-5D-5L mobility, self-care, usual activities, pain/discomfort subscales (all p-values >0.05) of McHeELP-G, except the Euro QoL-5D-5L anxiety/depression subscale (p=0.008) (Table 4). No statistically significant difference was observed in all qualitative outcomes of CP (p>0.05) (Table 4).

**Table 4:** Comparison of clinical qualitative outcomes’ measurements between groups Significant p-value < 0.05 * Post-intervention = end of the 3rd month (13th-week post-intervention) McHeELP: Motor Control Home Ergonomics Elderlies' Prevention of Falls

	McHeLP Group (n=10)			Control Group (n=10)		
Clinical qualitative variables	Baseline (n,%)	Post-intervention* (n,%)	p-value	Baseline (n,%)	Post-intervention* (n,%)	p-value
Knee flexion restriction						
Yes	4 (40%)	4 (40%)	1.000	–	–	1.000
Νο	6 (60.0%)	6 (60.0%)		10 (100%)	10 (100%)	
Knee extension restriction						
Yes	2 (20%)	2 (20%)	1.000	2 (20%)	2 (20%)	1.000
Νο	8 (80%)	8 (80%)		8 (80%)	8 (80%)	
Ankle dorsiflexion restriction						
Yes	2 (20%)	2 (20%)	1.000	2 (20%)	2 (20%)	1.000
No	8 (80%)	8 (80%)		8 (80%)	8 (80%)	
Ankle plantarflexion restriction						
Yes	–	–	1.000	–	–	1.000
No	10 (100%)	10 (100%)		10 (100%)	10 (100%)	
Euro QoL-5D-5L – Mobility subscale (min=1, max=5)						
2: I have slight problems in walking about	1 (10.0%)	1 (10.0%)	1.000	1 (10.0%)	1 (10.0%)	1.000
3: I have moderate problems in walking about	8 (80.0%)	8 (80.0%)		7 (70.0%)	7 (70.0%)	
4: I have severe problems in walking about	1 (10.0%)	1 (10.0%)		2 (20.0%)	2 (20.0%)	
Euro QoL-5D-5L – Self-care subscale (min=1, max=5)						
1: I have no problems washing or dressing myself	6 (60.0%)	6 (60.0%)	1.000	4 (40.0%)	2 (20%)	0.180
2: I have slight problems washing or dressing myself	3 (30.0%)	3 (30.0%)		4 (40.0%)	5 (50%)	
3: I have moderate problems washing or dressing myself	1 (10.0%)	1 (10.0%)		2 (20.0%)	3 (30%)	
Euro QoL-5D-5L – Usual activities subscale (min=1, max=5)						
1: I have no problems doing my usual activities	1 (10.0%)	1 (10.0%)	1.000	–	–	1.000
2: I have slight problems doing my usual activities	4 (40.0%)	4 (40.0%)		4 (40.0%)	4 (40%)	
3: I have moderate problems doing my usual activities	5 (50.0%)	5 (50.0%)		6 (60.0%)	6 (60 %)	
Euro QoL-5D-5L – Pain/ Discomfort subscale (min=1, max=5)						
1: I have no pain or discomfort	–	3 (30%)	0.086	–	–	1.000
2: I have slight pain or discomfort	3 (30.0%)	4 (40%)		4 (40.0%)	4 (40.0%)	
3: I have moderate pain or discomfort	7 (70.0%)	3 (30%)		6 (60.0%)	6 (60.0%)	
Euro QoL-5D-5L – Anxiety/ Depression subscale (min=1, max=5)						
1: I am not anxious or depressed	1 (10.0%)	1 (10%)	0.008	–	–	0.564
2: I am slightly anxious or depressed	1 (10.0%)	5 (50%)		4 (40.0%)	2 (20%)	
3: I am moderately anxious or depressed	5 (50.0%)	4 (40%)		5 (50.0%)	7 (70%)	
4: I am severely anxious or depressed	3 (30.0%)	–		1 (10.0%)	1 (10%)	

Regarding the future McHeELP RCT, it was calculated that a sample size of three patients per group was required in order to have a 90% probability of demonstrating a between-treatment difference of >15% (McHeELP-G: -34%±6 versus CG: -1%±6) in percentage change from baseline to the end of the intervention (end of the third month) of the TUG variable with a significance of <5% (two-tailed test). On the contrary, it was calculated that a sample size of 25 evaluable patients per group is required in order to have a 90% probability of demonstrating a between-treatment difference of > 15% (McHeELP-G: 32%±16 versus CG: 17%±16) in percentage change from baseline to the end of the intervention (end of the third month) of the Tandem variable with a significance of <5% (two-tailed test). Therefore, the Tandem test was set as the main study's sample size estimation variable, not the TUG test due to the minor required number of participants.

## Discussion

In the present pilot trial, the feasibility and acceptability of a novel intervention combining motor control home-based exercises and an ergonomic home safety-improvement strategy (the McHeELP program) were examined. In addition, the selections of the most appropriate primary and secondary outcomes, which must be used in the main McHeELP study (RCT), were analyzed.

As expressed by the selected PPMs and PROs, the pilot trial's results are indicative and supportive of our primary hypothesis that the McHeELP program will improve functional capacity and balance while reducing potential fall-risk factors in the home environment and the fear of falling in older adults. Exercise has been described as the most cost-effective strategy for preventing falls and fall-related fractures [8]. In a recent systematic review that includes 108 RCTs with 23,407 community-dwelling older adults from 25 countries, it was reported that exercise programs that reduce falls primarily involve balance and functional exercises while programs that probably reduce falls include multiple exercise categories (typically, balance and functional exercises plus resistance exercises) [1]. In 78 of those RCTs, the intervention arms included balance, coordination, gait, and functional task training [1]. Research findings regarding motor control exercises and falls are limited, although motor performance deficits in older adults include difficulty in coordination, increased movement variability, slowing of movement, and balance and gait difficulties [8]. We anticipate that the McHeELP program may give a novel look at healthy aging and the prevention of falls.

The McHeELP exercise program was created to be adjustable to an individual's skills while it can be performed both efficiently and safely at home without the need for supervision. The exercises were divided into six domains [1] (warm-up) and five motor control domains] [8]. The physiotherapist evaluated the functional level of each individual to tailor the best possible exercises from the McHeELP package. Each tailored program includes 12 exercises (two exercises from each of the six domains). All instructions for safe exercise prescription in the elderly were followed [8]. The exercises' progression was based on the number of repetitions, the patient's self-perceived fatigue, abilities and/or observed movement control, and the functional level of participants. For the sets of repetitions, the American College of Sports Medicine Position Stand guidelines for exercise and activity in older adults were taken into account [24]. Each participant received a detailed booklet that outlined their specific exercises. Every exercise was described on a separate page; representative and illustrative photographs accompanied the exercise's verbal description to ensure correct and safe performance without supervision. In order to maintain adherence, ongoing support, motivation, and proper progression of exercises, at the end of the first, fourth, and eighth weeks, the physiotherapist visited the participants, revised the individuated program, and made corrections and progressive adjustments. The booklet's pages were properly changed so that each timeframe included the 12 exercises that should be performed until the next appointment. This delivery method ensured that with this simplified implementation of the program, participants would be able to carry out the exercises tailored for them at each stage of the study with ease.

The excellent acceptability of McHeELP's program was confirmed by the 100% participant adherence, as is shown in the flow diagram of the study. The main reason could be that the exercises involved in our program were not very demanding physically and were chosen according to the individual's functionality, health status, and stated goals, without additional special and expensive equipment and/or technology expenditures. During the three-month intervention phase, no patient of McHeELP-G experienced severe pain or fatigue during the physiotherapy sessions. This could be explained by the fact that the selection of the proper McHeLP exercises was made by senior physiotherapists specialized in geriatric populations who were able to evaluate each participant's functional ability. Moreover, it has been reported that personalized home-based exercise programs may optimize the effectiveness of training, help lower the risk of falls, improve the physical performance of older adults, and increase the pleasure of exercising [25]. Another possible reason for the acceptability of McHeELP might be the fact that this pilot trial was performed after the first lockdown in Greece due to the COVID-19 pandemic, so the participants felt safer exercising at home rather than engaging in a group exercise program in physiotherapy units or other specialized centers for older adults.

To our knowledge, McHeELP is the first intervention approach that combines a home-based motor control exercise intervention with home assessment and modification. The McHeELP program, based on exercises targeting balance control systems, follows the principle that "training specificity is a key element of motor learning" [26]. Specifically, it has been reported that a task-oriented motor learning intervention may promote greater gains in clinical outcomes as compared to standard exercise programs in older adults [27]. In addition, the focus of the exercise on small adjustments is made for changes in the limbs, muscles, and posture, enabling the skilled mover to focus on the target of the movement while spinal and supraspinal motor centers organize and implement successful movement strategies [27]. As expressed by the selected PPMs and PROs, the post-intervention results showed increased functionality, balance control, and better health; hence, there are some potential beneﬁts of the implementation of the McHeELP exercises.

All outcome measures were collected comprehensively and accurately at the baseline and follow-up measures. We decided that the interview survey and PROs interspersed with the PPMs (i.e. demographic variables- 4 meters walking test - lifestyle habits - TUG test - LEFS-Greek, and so forth). This process allowed sufficient resting time between the tests and reduced the risk of question-order bias. The PROs were given out in random order and PPMs were performed only once, so as to minimize habituation bias and avoid affecting the participant's performance.

It was not surprising that no statistically significant improvement was found post-intervention, regarding the knee flexion/extension and the ankle dorsiflexion/plantarflexion restrictions since joint-flexibility exercises were not included in the McHeELP. According to this finding, it was decided that the joints' restrictions should be used as a supplementary clinical baseline measurement to detect any limited range of motion that could affect the proper performance of McHeELP's exercises or the performance of PPMs. We set the lower acceptable knee flexion limit at 105^o^, the knee extension at 10^o^, the ankle dorsiflexion, and the plantarflexion at 18^o^ and 58^o^, respectively. These ranges are adequate for the performance of the PPMs, including the TUG test, the FRT test, and the Sit-to-Stand test [22].

It is worth noting that a post-intervention statistically significant difference was observed in the FES-I_GREEK and EQ5D-5L anxiety/depression subscale (p=0.001 and p=0.008, respectively) of McHeELP-G. These findings are an indication that the McHeELP program may be adequate to reduce the anxiety and the concerns about fall-related self-efficacy. In order to further investigate this issue, it was decided that participants in the future McHeELP-RCT will be asked to assess their potential "fear of falling" using the Euro QoL-5D-5L anxiety/depression subscale. Another issue that has to be addressed about the future McHeELP-RCT is the results of the heel-to-shin test since no statistically significant difference was found between groups. A possible reason may be that the heel-to-shin test may be abnormal if there is a great loss of motor strength, proprioception, as in a cerebellar lesion. Since one of our exclusion criteria was lower-limb muscle weakness due to central or peripheral neurological etiology, perhaps this PPM may not be appropriate for our sample. Nevertheless, it was decided to keep it as a PPM of our future RCT; additionally, another lower limb proprioception test, developed by Lord et al. (2016) [28], will be added in order to examine the benefits of the McHeELP program in proprioception by using a lower limb-matching task. The Lord test is performed as follows: individuals are seated on a chair without armrests. A vertical transparent acrylic sheet (60×60×1 cm) inscribed with a protractor is placed between their legs. They are asked to align their lower limbs simultaneously on either side of the acrylic sheet-protractor, keeping their eyes closed. Any difference in aligning the lower limbs (indicated by disparities in matching the great toes on either side of the acrylic sheet-protractor) is measured in degrees. After two practice trials, an average of five experimental trials is recorded. Each trial is undertaken relatively quickly, with rests between trials, to avoid weakness unduly influencing the results [28].

Moreover, it was observed that no patient of both groups experienced a new fall-incident before the date of the second measurement (end of the third month). Considering this observation, we decided to record the numerous fall-incidents at the end of the third month (post-intervention) and the end of the sixth month (follow-up) measurements.

Our intervention also included home assessment and modifications. In the third-month measurement, as shown in the HOMEFAST results, a statistically significant difference was revealed in both McHeELP-G and CP (p=0.002 and p<0.001, respectively). The result was expected for McHeELP-G but not for CG. A possible explanation may be that all participants were fallers (inclusion criterion) and the McHeELP booklet's proposed home environment modifications are a low-cost adjustment. Thus, the CG participants realized the importance of that advice and, on their own, made their home environment less hazardous. According to this finding, it was reported that it is important for older adults to know how to prevent falls in their own residence and to be able to modify the environmental hazards in their home in order to maintain their daily residential safety [29].

The CG participants received no exercises during the study's timeframe and were instructed not to engage in any additional exercises for this period (12 weeks). However, a statistically significant difference was found between the post-intervention and baseline values of the Tandem Stance test (p=0.003). Considering that experienced and independent examiners performed the measurements, and no other outcome measure of GG had shown any significant difference, we theorize that this was a random result. Overall, this finding has not influenced our preliminary decision to use the Tandem Stance test as the variable for the sample size estimation of the future RCT study because it is a PPM used in older adults as an indicator of increased risk of fall [14].

Finally, in this pilot trial, the exact sample size that should be used for the future RCT was calculated. Considering the Tandem variable's sample size estimation (N=25) and the fact that 20% more participants are required in order to accommodate confounding factors, such as withdrawals, missing data, and lost to follow-up [30], it was decided that 30 older adults per group (a total sample of 60 participants) need to participate in the main McHeELP study.

Strengths and limitations

This study conformed to the "Consolidated Standards of Reporting Trials" (CONSORT) extension statement for randomized pilot and feasibility trials [10]. Supervision and guidance from the physiotherapist during sessions helped ensure the participant's adherence. Furthermore, all measurements were made by the same examiner, who was not involved in any part of the McHeELP program and was blinded with respect to the group assignment. These factors added strength and statistical power to the results of this pilot trial. On the other hand, there are important limitations that must be mentioned. A limitation of the present study is that the small sample size needed for a pilot study precludes broad generalizations. The sample size is an important consideration when a clinical trial is planned for the main trial and for any preliminary results. However, the main goal is to try out aspects of the proposed main trial [23]. In addition, in the present study, our sample did not include a relatively diverse sample. The demographic characteristics of intervention and control participants were similar. Finally, a possible risk of bias may be that the selection of appropriate exercises for the individualized home-based program was made by highly experienced physiotherapists in the geriatric population, a fact that may influence the compliance of the participants and the excellent acceptability of the McHeLP program.

## Conclusions

The present pilot trial demonstrated that the McHeELP program, a novel home-based and low-cost intervention, was feasible and acceptable to participants. The results shown here indicate that McHeELP's implementation may enhance functional capacity and improve balance in community-dwelling older adults; thus, it was concluded that it is practical and essential to deliver an RCT. Nevertheless, further research is needed to understand the effects of this targeted exercise program on older adults' motor control beyond our future RCT. Easy-to-administer exercise programs need to be developed and implemented to reduce the burden of falls in older adults.

## Appendices

**Table 5:** Description and procedures of the McHeELP pilot trial’s outcome measures McHeELP: Motor Control Home Ergonomics Elderlies' Prevention of Falls; PPM: physical performance measure; VAS: visual analog scale

Outcome measures	Description and procedures
4 meters walking test [11]	Objective: It is a PPM that is used to assess functionality and physical performance in older adults. Test procedure: Participants are informed to walk 4 m at their usual speed. Walking speed is assessed in seconds by a manual chronometer. Timing starts at the first foot movement and ends when a foot completely crosses the finish line Test findings: Walking speed is recorded in seconds.
Timed Up and Go test [12]	Objective: This test determines fall risk and measures the ability to balance, sit to stand, and walking. Test procedure: Participants are seated in a standard 45 cm height chair, with the back against the chair, both arms resting along their body and both feet completely resting on the floor. The TUG assesses the number of seconds needed for an individual to stand up from a chair, walk 3 meters at their usual pace past a line on the floor, turn around, walk back to the chair, and sit down again with the back against the chair. Test findings: Performance time is recorded in seconds.
Sit to stand test (30 sec) [13]	Objective: It is a PPM for testing leg strength and endurance in older adults. Test procedure: The 30-second sit-to-stand test records the number of stands a person can complete in 30-seconds. The test is administered using a chair without arms, with a seat height of 43 cm (17 inches). Test findings: The number of stands is recorded.
Tandem stance test (heel-toe) [14]	Objective: It is a PPM of standing balance testing postural steadiness in a heel-to-toe position. Test procedure: The test requires the participant to maintain balance while standing in a tandem heel-to-toe position. The participant places the foot immediately in front of the other foot (heel to toe), arms down by their side. Test findings: the time that the participant is able tohold the tandem stance is recorded in seconds.
Functional Reach Test (FRT) [15]	Objective: This is an assessment tool for ascertaining dynamic balance in one simple task. Test procedure: The participant barefoot and standing upright, is positioned with one side (e.g. right) of the body close to the wall. A yardstick is attached to a wall at about shoulder height. The instruction is to “reach forward along the yardstick as far as you can without taking a step” along the yardstick. The location of the 3rd metacarpal is recorded. Test findings: The researcher measures the distance the person can reach forward beyond the arm length while standing in a fixed position. [15]
Foot tapping test (10 sec) [16]	Objective: It is used as a clinically useful measure to quantify the slowness of voluntary leg movements. Test procedure: The participant sits on a chair with its height adjustable, so that the bilateral soles made contact with the floor, with the hip and knee joints flexed at approximately 90°. He/she moves their toes up and down repeatedly, tapping the floor as quickly and vigorously as possible, with their heels firmly planted on the floor for 10 sec. Test findings: The researcher counts the number of taps.
Heel to shin test [17]	Objective: It is a test of lower limb coordination and position sense, often performed to assess the coordination and maybe abnormal if there is loss of motor strength, proprioception, or a cerebellar lesion. Test procedure: The patient is asked to lay supine and place his/her right heel on their left shin just below the knee and then slide it down their shin to the ankle, repeating as quickly and as accurately as possible. Have the patient repeat this movement with the other foot. Test findings:Assess for accuracy of heel placement. Note if the heel is not pushed down the shin smoothly and accurately.
Euro QoL-5D-5L questionnaire – Greek version [18]	Objective: The EQ-5D-5L is a well-known and widely used health status instrument; measuring health-related quality of life. The EQ-5D-5L assesses five dimensions of health (mobility, self-care, usual activity, pain/discomfort, and anxiety/depression. The second part of the questionnaire used the VAS Scale and asks responders to self-rate themselves on a “thermometer” scale. Score findings (min – max): Euro QoL-5D-5L – Subscales: The descriptive system comprises the following five dimensions: mobility, self-care, usual activities, pain/discomfort, and anxiety/depression, each with five levels: a level 1 response represents “no problems,” level 2 “slight problems,” level 3 “moderate problems,” level 4 “severe problems,” and level 5 “extreme problems” or “unable to perform,” which is the worst response in the dimension. Euro QoL-5D-5L – VAS: the endpoints are labeled 0 = the worst health you can imagine and 100 = the best health you can imagine.
Lower Extremity Functional Scale – Greek version (LEFS- Greek) [19]	Objective: The LEFS is a 20-item questionnaire functional status questionnaire that aims to assess functional status and investigates the degree of difficulty a patient experiences in performing everyday tasks, in patients with disability of the lower extremity. Score findings (min – max): min=0 (very low functionality) – max =80 (very high functionality)
Falls self-efficacy international scale – Greek version (FES-I_GREEK) [20]	Objective: This scale was developed by members of the Prevention of Falls Network Europe (ProFaNE) consensus group. FES-I, a 16-item questionnaire has been widely used for assessing the fear of falling. Score findings (min – max): min=16 (no concerns about fall-related self-efficacy) – max=64 (severe concerns about fall-related self-efficacy)
Home falls and accidents screening tool questionnaire – Greek version (HOMEFAST) [21]	Objective: The HOMEFAST is a questionnaire is used to identify hazards in older adults’ homes. It consists of 25 items focusing on seven main areas of potential hazards: floors, furniture, lighting, bathroom, storage, stairways/steps, and mobility. Score findings (min – max): min=0 (no risk of falling within the home environment) – max=25 (higher risk of falling within the home environment)
